# Efficacy and safety of obinutuzumab in patients with primary membranous nephropathy: a focus on refractory and rituximab-resistant patients

**DOI:** 10.3389/fimmu.2026.1825745

**Published:** 2026-06-26

**Authors:** Qi Gao, Guoxiang Yao, Yujiao Sun, Xiaoyun Li, Zhuo Li, Bing Chen

**Affiliations:** Department of Nephrology, Shandong Provincial Hospital Affiliated to Shandong First Medical University, Jinan, Shandong, China

**Keywords:** clinical remission rate, kidney, obinutuzumab, PLA2R antibody, primary membranous nephropathy

## Abstract

**Background:**

There has been an annual rise in the incidence of primary membranous nephropathy (PMN), a common pathological type of nephrotic syndrome (NS). Obinutuzumab is a humanised, glycosylated, type II anti-CD20 monoclonal antibody. Characterised by its enhanced B-cell depletion capability compared with rituximab (RTX), obinutuzumab has demonstrated promising efficacy in the treatment of membranous nephropathy.

**Objective:**

This study aimed to investigate the efficacy and safety of obinutuzumab in treating various subtypes of PMN.

**Methods:**

A total of 94 PMN patients with NS were enrolled in this study. Based on their treatment history before receiving obinutuzumab, the patients were categorised into the Initial therapy group (group A, n=32), the Refractory group (group B, n=32), and the Remedial therapy group (group C, n=30). The efficacy and safety of obinutuzumab treatment after a 24-month follow-up period were observed and analysed in each group. The primary endpoint was the clinical remission rate (complete remission + partial remission) at 24 months, and the secondary endpoints were treatment safety and the incidence of treatment-emergent adverse events.

**Results:**

Following 24 months of obinutuzumab therapy, 95.74% (90/94) of the PMN patients achieved clinical remission, with 28.72% (27/94) reaching complete remission (CR) and 67.02% (63/94) achieving partial remission (PR). The Initial therapy group (group A) achieved a 100% (32/32) clinical remission rate: 37.5% (12/32) attained CR, and 62.5% (20/32) attained PR. The Refractory group (group B) achieved a 96.88% (31/32) clinical remission rate: 25% (8/32) attained CR, and 71.88% (23/32) attained PR. The Remedial therapy group (group C) achieved a 90% (27/30) clinical remission rate: 23.33% (7/30) attained CR, and 66.67% (20/30) attained PR. All 63 anti-PLA2R antibody-positive (>20 U/mL) patients and 16 anti-PLA2R antibody negativity but no immunological remission (2–20 U/mL) patients demonstrated declining antibody levels following obinutuzumab therapy, and 68.4% (54/79) attained complete immunological remission (<2 U/mL).

**Conclusion:**

Obinutuzumab demonstrates high clinical remission rates and acceptable safety in PMN patients, with good efficacy even in refractory and RTX-resistant patients.

## Introduction

Primary membranous nephropathy (PMN) is an immune complex-mediated kidney disease and a common cause of primary glomerulonephritis in adults. It is pathologically characterised by diffuse thickening of the glomerular basement membrane and the presence of subepithelial immune deposits, primarily composed of IgG and C3. Clinically, it most commonly presents with nephrotic syndrome (NS) or asymptomatic proteinuria (1, 2). The disease course is highly variable, ranging from spontaneous remission to persistent proteinuria and progression to end-stage kidney disease (ESKD) (3, 4). Approximately one-third of patients achieve spontaneous remission (5), whereas 40%–50% of untreated patients with persistent NS develop ESKD. Therefore, proactive intervention and treatment for patients with PMN are crucial in delaying the progression of kidney disease.

The pathogenesis of PMN, an immune-mediated disease confined to a single organ, is primarily driven by autoantibodies targeting podocyte antigens such as the M-type phospholipase A2 receptor (PLA2R) (6) and thrombospondin type-1 domain-containing 7A (THSD7A) (7, 8). By targeting podocytes and activating the complement system, these antibodies cause sublethal cell injury, which, in turn, impairs the function of the glomerular filtration barrier and induces proteinuria (9). These significant breakthroughs demonstrate that PMN is an autoimmune disease targeting podocytes and that antibodies produced by autoreactive B-cell clones are the key initiating factor in the pathogenesis of membranous nephropathy (MN). This provides compelling evidence for B-cell-targeted therapies in PMN.

The 2012 Kidney Disease: Improving Global Outcomes (KDIGO) Clinical Practice Guidelines for Glomerular Diseases recommend immunosuppressive therapy with glucocorticoid rescue therapy combined with alkylating agents (most commonly cyclophosphamide (CTX)) as the first-line treatment (10). Alkylating agents have been shown to improve NS and slow the progression of kidney disease compared with non-immunosuppressive therapies. However, in clinical practice, glucocorticoid rescue therapy with alkylating agents is associated with significant toxic side effects, including severe infections, bone marrow suppression, late-onset malignancies, infertility, and other serious adverse events (11). The guideline-recommended treatment options also include calcineurin inhibitors (CNIs) such as cyclosporine A (CsA) and tacrolimus (TAC). However, the use of CNIs is limited by a high relapse rate of 40%–50% and the risk of nephrotoxicity (12, 13).

Rituximab (RTX) appears to be a safer alternative with a high clinical remission rate. It is a human–mouse chimeric monoclonal antibody that specifically targets the CD20 antigen on B cells. RTX induces B-cell death through apoptosis, complement-dependent cytotoxicity (CDC), and antibody-dependent cellular cytotoxicity. It can avoid the adverse effects associated with glucocorticoids and traditional immunosuppressants, making it an effective drug for the treatment of MN (14). Since its first reported use in 2002 for treating patients with PMN, RTX has emerged as a preferred treatment option due to its high safety and efficacy profile. A clinical trial investigating RTX for MN, known as the MENTOR study, demonstrated that patients receiving RTX achieved a proteinuria remission rate of 66.7% with a favourable safety profile. Consequently, RTX has become a first-line treatment for non-severe PMN. However, it is associated with a relatively high relapse rate (27%), necessitating repeated administration, and 35%–45% of cases show no response (15, 16). Consequently, the number of reported cases of RTX-resistant MN has risen. The analysis suggests that contributing factors may include inadequate RTX dosage (associated with increased urinary loss due to heavy proteinuria and internalisation/degradation of RTX by B cells), the production of anti-RTX antibodies (linked to increased urinary protein, elevated PLA2R-Ab titres, and higher relapse rates in MN patients), chronic irreversible glomerular damage, and epitope spreading (17). This has prompted the introduction of novel biological agents involving different molecular mechanisms. For the treatment of PMN, finding superior alternatives to conventional therapies—with lower relapse rates and improved safety profiles—represents an urgent and critical clinical goal.

Recent studies indicate that while RTX primarily targets the peripheral circulation, obinutuzumab can penetrate further into lymph nodes. The elimination “depth” progressively increases with T-cell engagers, CAR-T cell therapies, and allogeneic stem cell transplantation technologies. Obinutuzumab alleviates PMN by specifically binding to the CD20 antigen on B cells, depleting CD20-positive B cells (18). This reduction in B cells decreases circulating antibody production, thereby preventing the formation of immune deposits beneath the glomerular epithelium and mitigating damage to the glomerular filtration barrier (19). Obinutuzumab is engineered through glycoengineering and other modifications to its Fc region, which enhance the antibody’s binding affinity for FcγRIIIa and thereby augment its effector functions, ultimately leading to improved therapeutic activity. Obinutuzumab is a more effective B-cell depleting agent than type I anti-CD20 monoclonal antibodies such as RTX (20). The primary mechanisms include (1) glycoengineering: this exhibits a 35-fold enhancement in antibody-dependent cellular cytotoxicity (ADCC) compared with RTX and (2) type II binding conformation: this results in reduced internalisation into lipid rafts, enhanced stability, and an increased capacity to induce direct cell death. Consequently, obinutuzumab demonstrates stronger ADCC, superior direct B-cell killing, reduced dependence on CDC, and lower immunogenicity risk (20, 21). These characteristics endow it with potential advantages in patients who have failed or are intolerant to RTX. In recent years, multiple case reports and small-scale studies have suggested that obinutuzumab may be effective in refractory PMN and RTX-resistant patients. However, systematic studies targeting PMN patients with diverse treatment backgrounds (particularly refractory and RTX-unresponsive cases) remain limited.

This study aims to evaluate the efficacy and safety of obinutuzumab in the treatment of patients with primary membranous nephropathy through a retrospective analysis, with the goal of providing more evidence-based guidance for the clinical management of refractory cases and those with poor response to rituximab.

## Materials and methods

### Study participants

#### 1 Study design and setting

This study was a retrospective cohort study conducted in the Department of Nephrology, Shandong Provincial Hospital Affiliated to Shandong First Medical University. This study complied with the Declaration of Helsinki and was approved by the Ethics Committee of Shandong Provincial Hospital (JNKJ: NO.2020-3028). In accordance with national legislation and institutional requirements, written informed consent for participation was not required for this study due to its retrospective nature and the use of anonymised data.

#### 2 Study population and consecutive enrolment

Consecutive adult patients (aged >18 years) with biopsy-proven primary membranous nephropathy (PMN) who were treated at our hospital between January 2021 and January 2024 were screened through the electronic medical record (EMR) system. Initially, 120 patients who received obinutuzumab therapy were screened. Based on the inclusion and exclusion criteria, 26 patients were excluded: 15 received non-standard dosing and 11 had a follow-up duration of less than 24 months. Consequently, 94 patients were included in this retrospective analysis.

#### 3 Inclusion and exclusion criteria

Inclusion criteria were as follows: (1) the patients were diagnosed with PMN by renal biopsy at our hospital; (2) before obinutuzumab treatment, the patients presented with clinical features of NS, defined as 24-h urinary protein excretion >3.5 g/24 h, serum albumin <30 g/L; (3) obinutuzumab was administered as either initial or remedial therapy; (4) the patients had complete clinical and laboratory records and underwent a comprehensive evaluation—including medical history, physical examination, and serological/imaging studies—to rule out potential causes of secondary MN, such as underlying malignancies, pathogenic drug exposure, hepatitis B/C, HIV, and autoimmune diseases. Exclusion criteria were as follows: (1) patients who received non-standard dosing of obinutuzumab (n=15); (2) Patients with a follow-up duration of less than 24 months (n=11).

#### 4 Subgroup assignment and treatment decision process

Patient subgroup assignment was not pre-specified prior to treatment but was performed *post-hoc* by the investigators based on the complete treatment history prior to obinutuzumab therapy after data collection. The specific classification rules were as follows:

1. Initial therapy group (hereafter referred to as group A): patients who had not previously received any immunosuppressive agents. These patients had not received any immunosuppressive therapy prior to obinutuzumab treatment but had all received supportive care, including blood pressure control, reduction of urinary protein levels, and treatment with angiotensin-converting enzyme inhibitors or angiotensin II receptor blockers for at least 3 months yet remained in a persistent nephrotic syndrome (NS) state. During this period, blood pressure was maintained at no higher than 140/90 mmHg.
2. Refractory group (hereafter referred to as group B): patients who had previously received calcineurin inhibitors (CNIs) or cyclophosphamide (CTX) for at least 6 months without remission. These patients had received at least 6 months of an adequate course of conventional immunosuppressive therapy (CNIs or CTX) but had not achieved complete remission (CR) or partial remission (PR), defined as 24-h urinary protein still >3.5 g/24 h. After discontinuing previous immunosuppressive agents, they were switched to obinutuzumab therapy.
3. Remedial therapy group (hereafter referred to as group C): patients who had previously received rituximab (RTX) for at least 6 months without anti-PLA2R antibody seroconversion and were subsequently given obinutuzumab. These patients had received at least one standard full-course RTX regimen, and at least 6 months after discontinuation of RTX, their anti-PLA2R antibody levels remained >2 U/mL (i.e., did not achieve complete immunological remission) and they remained in a state of unremitted nephrotic syndrome. Obinutuzumab was then administered as remedial therapy.

### Clinical data

Basic information included gender, age, and time of onset. Laboratory tests included 24-h urinary protein quantification, urinalysis, urinary red blood cells, haemoglobin, white blood cell count, platelet count, lymphocyte count, serum total protein, serum albumin, total cholesterol, serum creatinine, blood urea nitrogen, glomerular filtration rate, serum anti-PLA2R antibody, CD19+ B-cell count, immunoglobulin G (IgG), immunoglobulin A (IgA), and immunoglobulin M (IgM). Follow-up data included follow-up duration, remission status, number of relapses, and adverse reactions. Follow-up laboratory tests included 24-h urinary protein quantification, urinalysis, serum albumin, serum creatinine, serum anti-PLA2R antibody, and CD19+ B-cell count.

### Treatment plan and follow-up

The administration regimen in this study was as follows: Obinutuzumab was administered intravenously using a two-dose protocol: 1 g infused on day 1, followed by another 1-g infusion after a 14-day interval. Obinutuzumab was diluted in 500 mL of 0.9% physiological saline to achieve a concentration of 1 mg/mL. The infusion was initiated at a rate of 40 mL/h and gradually increased up to 200 mL/h based on each patient’s tolerance. To minimise infusion-related reactions, the patients were premedicated with methylprednisolone 40 mg, dexamethasone 5 mg, and promethazine 25 mg before the obinutuzumab infusion. Follow-ups were conducted every 3 months, specifically before obinutuzumab treatment and at 3, 6, 9, 12, 15, 18, 21, and 24 months after treatment. Monitoring indicators at each visit included complete blood count, urinalysis, liver and kidney function, lipid profile, blood glucose levels, 24-h urinary protein quantification, anti-PLA2R antibody levels, and circulating B-cell count. We used a standardised commercial ELISA method (Euroimmun, Lubeck, Germany) to measure anti-PLA2R antibody titres, with a titre >20 U/mL defined as positive. B-cell depletion was defined as <5 B cells/mm³ in peripheral blood. Adverse events related to obinutuzumab during drug infusion and throughout the follow-up period were recorded. Subsequent follow-ups were conducted at 3-month intervals to document patient remission and relapse status.

For the above administration regimen, a decision on whether to administer an additional dose was made at 3 to 6 months post-treatment based on the patient’s 24-h urinary protein quantification, degree of B-cell recovery, anti-PLA2R antibody levels, and clinical remission status.

### Efficacy evaluation and renal outcomes

The primary endpoint was the clinical remission rate (complete remission + partial remission) of patients at 24 months. The secondary endpoints were the safety and incidence of adverse reactions following medication administration. According to the 2021 KDIGO guidelines (22), for the evaluation of treatment response, remission time was defined as the period from the first medication administration to the resolution of urinary protein. CR was defined as 24-h urinary protein <0.3 g with stable renal function (eGFR ≥45 mL/min/1.73 m²). PR was defined as 24-h urinary protein <3.5 g with stable renal function (eGFR ≥45 mL/min/1.73 m²), or a ≥50% reduction in 24-h urinary protein from baseline, along with a ≥30% increase or normalisation of serum albumin concentration, and stable serum creatinine (or an increase <30%). Patients who did not meet the above criteria were considered non-responders, meaning they did not achieve clinical remission. Relapse was defined as the reappearance of 24-h urinary protein quantification exceeding 50% of the baseline value or >3.5 g in patients who had previously achieved CR or PR. The primary endpoint for renal outcomes was the deterioration of renal function or the occurrence of ESKD. Deterioration of renal status was defined as a post-treatment increase in serum creatinine to >133 μmol/L or a sustained doubling of the baseline serum creatinine level for more than 3 months. ESKD was defined as a creatinine clearance rate below 15 mL/min at the last follow-up, initiation of dialysis, or kidney transplantation. Adverse reactions included osteoporosis, infections, gastrointestinal bleeding, electrolyte disturbances, hypertension, hyperglycaemia, cataracts, weight gain, induced generalised seizure onset, and psychiatric symptoms, among others. The most common adverse reactions associated with obinutuzumab were infusion-related reactions, including rash, erythema, pruritus, rhinorrhoea, and restlessness (23). Serious adverse events were defined as clinical death or the occurrence of conditions such as severe pulmonary infection, pulmonary embolism, cerebral infarction, myocardial infarction, or hospitalisation due to adverse events (23, 24).

### Statistical methods

Statistical analysis in this study was performed using SPSS 27.0. Continuous variables following a normal distribution were expressed as mean ± standard deviation, and intergroup comparisons were conducted using t-tests. Non-normally distributed continuous variables were presented as median (interquartile range), with intergroup comparisons performed using the Mann–Whitney U test. One-way analyses of variance (ANOVA) were applied for comparisons among multiple groups (≥3) of continuous variables. Categorical variables were expressed as frequencies, and intergroup comparisons were made using the chi-square test. A Cox regression model was used to analyse the predictors of achieving clinical remission within 24 months. All tests were two-tailed, with the significance level set at 0.05. A P-value <0.05 indicated statistical significance, whereas P <0.01 indicated high statistical significance.

## Results

### Baseline characteristics

This study considered a total of 94 patients with PMN, with a median age of 53.0 (37.0, 59.0) years, comprising 65 men and 29 women. Before treatment with obinutuzumab, the median 24-h urine protein level was 6.3 (4.0, 9.8) g/24 h, serum albumin was 24.7 ± 4.9 g/L, serum creatinine was 74.5 (58.8, 90.6) μmol/L, eGFR was 96.0 ± 24.3 mL/min/1.73 m², and anti-PLA2R antibody was 50.2 (8.1, 222.3) U/mL. In group C, patients who had received a full course of standard RTX treatment for ≥6 months had a median 24-h urine protein level of 4.1 (2.2, 7.1) g/24 h, serum albumin of 29.5 ± 5.3 g/L, serum creatinine of 65.8 ± 13.2 μmol/L, and anti-PLA2R antibody of 8.1 (2.4, 21.0) U/mL. These patients had not achieved complete antibody clearance (PLA2R antibody levels remained >2 U/mL) and were still in a state of unremitted NS. Subsequently, obinutuzumab rescue therapy was administered.

**Figure 1:**
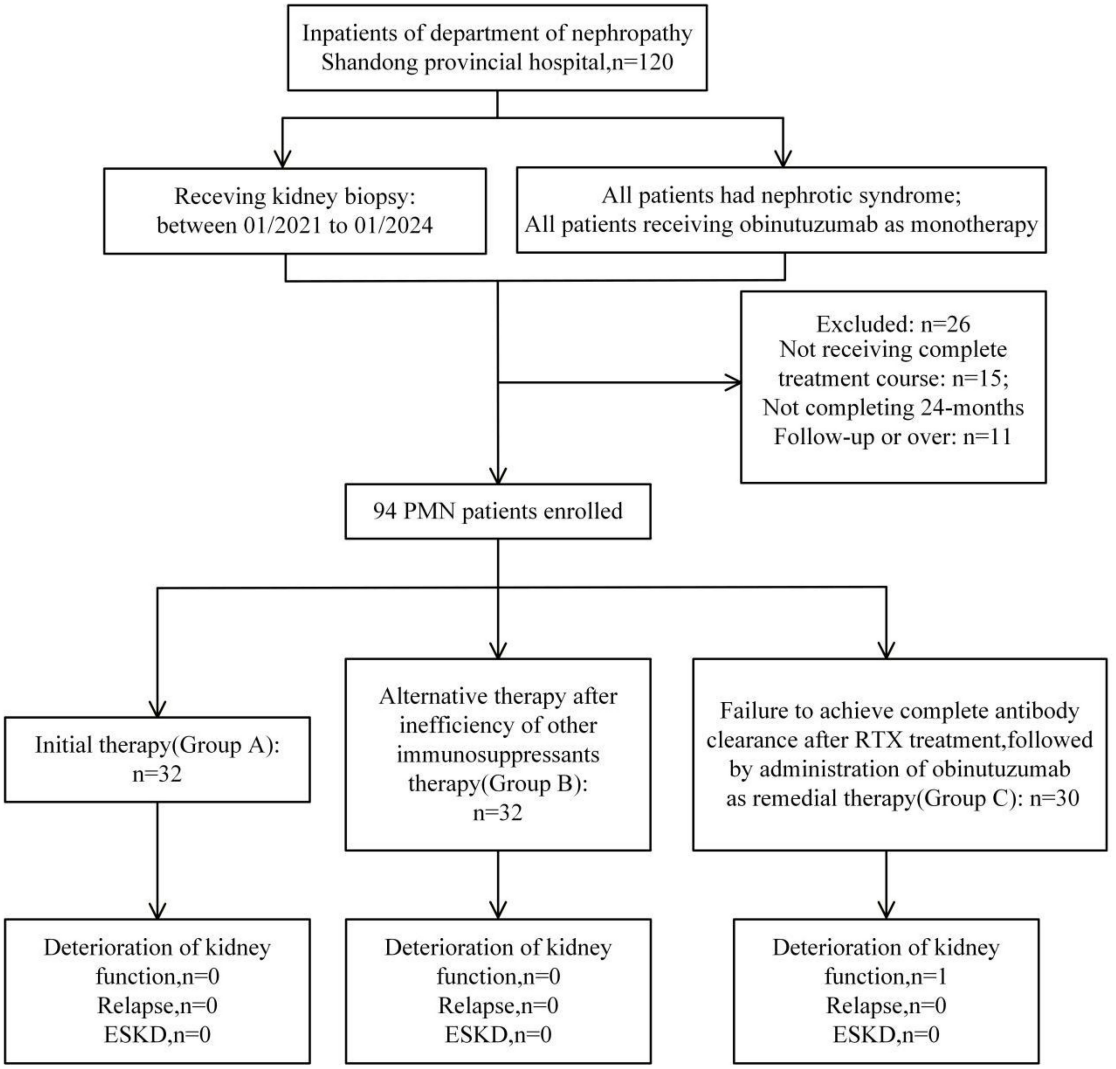
Flowchart of the patients with PMN receiving obinutuzumab therapy. There were 94 PMN patients enrolled, with 32 patients received obinutuzumab as initial therapy [group **(A)**], 32 as an alternative therapy after inefficiency of other immunosuppressants therapy [group **(B)**], and 30 as a remedial therapy after failure to achieve complete antibody clearance after RTX treatment [group **(C)**], followed by administration of obinutuzumab. During follow-up, one patient [from group **(C)**] had deterioration of kidney function, no patient relapsed. No patients entered ESKD.

Group A, group B, and group C comprised 32, 32, and 30 patients, respectively (see **Figure 1** for details). Patients in group C exhibited higher overall levels of 24-h urinary protein quantification [7.9 (5.6, 11.8) vs. 5.3 (4.0, 9.8) vs. 5.5 (3.8, 8.4) g/24 h, P = 0.026; the difference was statistically significant] than those in group A and group B. This indicates a relatively severe degree of renal injury in the patients in group C. Patients in group B exhibited lower levels of anti-PLA2R antibodies [8.1 (1.4, 41.5) vs. 76.4 (22.1, 173.7) vs. 86.9 (46.4, 339.0) U/mL, P = 0.001; the difference was statistically significant] and lower absolute CD19 levels [72.2 (5.8, 182.6) vs. 313.4 (162.9, 558.9) vs. 187.2 (168.5, 506.3) cells/μL, P = 0.004; the difference was statistically significant] than those in group A and group C. This indicates that conventional immunosuppressive therapy was effective but failed to achieve complete remission (CR) or partial remission (PR) in the patients in group B (for details, see **Table 1**). Among them, 88 patients had an eGFR >60 mL/min/1.73 m², whereas six patients had an eGFR between 45 and 60 mL/min/1.73 m².

**Table 1:** Baseline characteristics of patients with PMN considered in this study.

Characteristic	Total (n=94)	Group A (n=32)	Group B (n=32)	Group C (n=30)	P
Male sex, n(%)	65 (69.1%)	20 (62.5%)	24 (75%)	21 (70%)	0.552
Age (years)	53.0 (37.0, 59.0)	49.0 (37.8, 58.0)	56.0 (35.0, 63.5)	53.0 (42.3, 56.3)	0.585
Urine RBC/µL	3.8 (1.3, 9.7)	7.6 (2.4, 10.8)	3.0 (1.0, 9.5)	3.1 (1.5, 9.9)	0.395
Proteinuria (g/24 h)	6.3 (4.0, 9.8)	5.3 (4.0, 9.8)	5.5 (3.8, 8.4)	7.9 (5.6, 11.8)	0.026
WBC (×109/L)	6.5 (5.3, 8.4)	6.4 (5.2, 8.7)	6.5 (5.6, 7.0)	6.5 (4.9, 9.1)	1.000
Haemoglobin (g/L)	133 (115, 147)	138.0 (119.0, 152.5)	128.5 (111.5, 135.8)	133.0 (119.0, 147.3)	0.041
Platelet (×109/L)	255.7 ± 74.6	246.4 ± 69.1	249.5 ± 62.9	269.8 ± 91.1	0.426
AST (u/L)	21 (19, 24)	21.0 (19.5, 22.0)	20.5 (17.0, 23.3)	23.0 (14.0, 29.3)	0.155
ALT (u/L)	19 (16, 21)	19.0 (16.0, 20.0)	17.0 (12.5, 21.0)	21.0 (17.5, 24.0)	0.192
Total protein (g/L)	46.3 (42.9, 54.0)	46.0 (43.5, 50.8)	50.1 (43.2, 56.6)	45.3 (41.6, 49.5)	0.033
Albumin (g/L)	24.7 ± 4.9	24.0 ± 4.2	25.9 ± 5.5	23.7 ± 4.7	0.157
Globulin (g/L)	22.0 (19.9, 25.0)	22.5 (19.9, 25.5)	22.0 (19.9, 25.1)	22.0 (19.9, 24.0)	0.958
BUN (mmol/L)	7.0 ± 2.5	6.5 ± 1.8	7.2 ± 2.2	7.8 ± 3.2	0.343
Serum creatinine (μmol/L)	74.5 (58.8, 90.6)	73.9 (58.1, 94.2)	76.9 (56.2, 102.1)	74.5 (59.9, 85.1)	0.869
eGFR (mL/min/1.73 m2) a	96.0 ± 24.3	94.3 ± 22.9	94.9 ± 30.1	97.8 ± 19.0	0.841
GFR>60,n(%)	88 (93.6%)	30 (93.8%)	28 (87.5%)	30 (100%)	0.132
GFR45–60,n(%)	6 (6.4%)	2 (6.2%)	4 (12.5%)	0	0.132
Cholesterol (mmol/L)	6.5 (5.5, 8.2)	6.5 (5.4, 8.1)	6.0 (5.0, 6.9)	7.3 (6.1, 9.4)	0.095
Anti-PLA2R antibody positivity, n(%)b	63 (67.0%)	25 (78.1%)	13 (40.6%)	25 (83.3%)	0.001
Anti-PLA2R antibodies (>150U/mL)	25 (26.6%)	8 (25%)	5 (15.6%)	12 (40%)	0.001
Anti-PLA2R antibodies (U/mL)	50.2 (8.1, 222.3)	76.4 (22.1, 173.7)	8.1 (1.4, 41.5)	86.9 (46.4, 339.0)	0.001
The absolute values of CD19 (/μL)	180.6 (74.0, 354.5)	313.4 (162.9, 558.9)	72.2 (5.8, 182.6)	187.2 (168.5, 506.3)	0.004

Values are presented as number (%), median (interquartile range), or mean ± SD.

PMN, idiopathic membranous nephropathy; ALT, glutamic pyruvic transaminase; AST, glutamic oxaloacetic transaminase; WBC, white blood cell; BUN, blood urea nitrogen; eGFR, estimated glomerular filtration rate; PLA2R, phospholipase A2 receptor; IgA, immunoglobulin A.

a. eGFR was calculated according to the Chronic Kidney Disease Epidemiology Collaboration equation.

b. Anti-PLA2R positivity was defined by a value>20 U/mL.

### Efficacy evaluation

**Figure 2:**
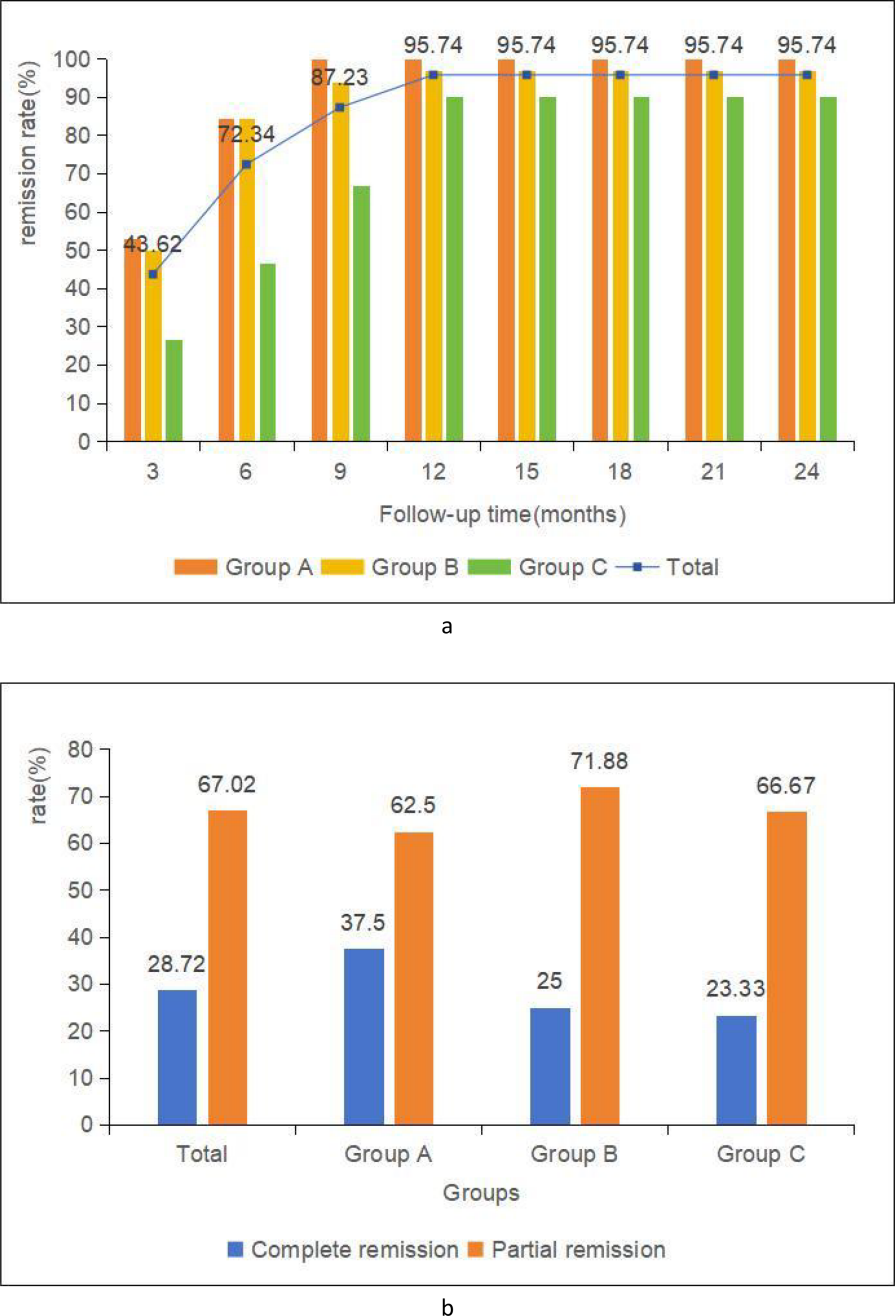
Trends in clinical remission (CR+PR) rates during the 24-month follow-up period. The primary outcome was clinical remission at 24 months. The remission rate was 95.74% (90/94) in total. Group C had a lower remission rate compared with group A and group B at 24 months, but the difference was not statistically significant (P = 0.118).

All the patients in this study completed a minimum follow-up period of 24 months. At the 24-month mark, the overall response rate was 95.74%, with 27 patients (28.72%) achieving CR and 63 patients (67.02%) achieving PR. In group A, the overall response rate was 100%, with 12 cases (37.5%) achieving CR and 20 cases (62.5%) achieving PR. In group B, the overall response rate was 96.88%, with 8 cases (25%) achieving CR and 23 cases (71.88%) achieving PR. In group C, the overall response rate was 90%, with seven cases (23.33%) achieving CR and 20 cases (66.67%) achieving PR (for details, see **Table 2**; **Figure 2**).

**Table 2:** Complete remission (CR) and clinical remission (CR+PR) rates from month 3 to month 24.

Study time points	No. of patients with remission/total no. (%)				P
	Total (n=94)	Group A (n=32)	Group B (n=32)	Group C (n=30)	
Complete remission					
3 months	10 (10.64%)	6 (18.75%)	2 (6.25%)	2 (6.67%)	0.186
6 months	21 (22.34%)	11 (34.38%)	6 (18.75%)	4 (13.33%)	0.116
9 months	25 (26.60%)	12 (37.5%)	8 (25%)	5 (16.67%)	0.173
12 months	27 (28.72%)	12 (37.5%)	8 (25%)	7 (23.33%)	0.397
18 months	27 (28.72%)	12 (37.5%)	8 (25%)	7 (23.33%)	0.397
24 months	27 (28.72%)	12 (37.5%)	8 (25%)	7 (23.33%)	0.397
Complete or partial remission					
3 months	41 (43.62%)	17 (53.13%)	16 (50%)	8 (26.67%)	0.074
6 months	68 (72.34%)	27 (84.38%)	27 (84.38%)	14 (46.67%)	0.001
9 months	82 (87.23%)	32 (100%)	30 (93.75%)	20 (66.67%)	0
12 months	90 (95.74%)	32 (100%)	31 (96.88%)	27 (90%)	0.118
18 months	90 (95.74%)	32 (100%)	31 (96.88%)	27 (90%)	0.118
24 months	90 (95.74%)	32 (100%)	31 (96.88%)	27 (90%)	0.118

The primary outcome was clinical remission (CR+PR) at 24 months. Results at earlier time points (3, 6, 9, 12, and 18 months) are shown for descriptive purposes only.

During the follow-up period of this study, the clinical remission rate of the patients gradually increased with the extension of follow-up time. Group C showed a lower remission rate than groups A and B (90% vs. 100% vs. 96.88%, P = 0.118; the difference was not statistically significant). The difference in clinical remission rates among the three groups became significant as early as the 3-month mark (26.67% vs. 53.13% vs. 50%) and remained consistent throughout the study period.

### Changes in laboratory parameters

**Figure 3:**
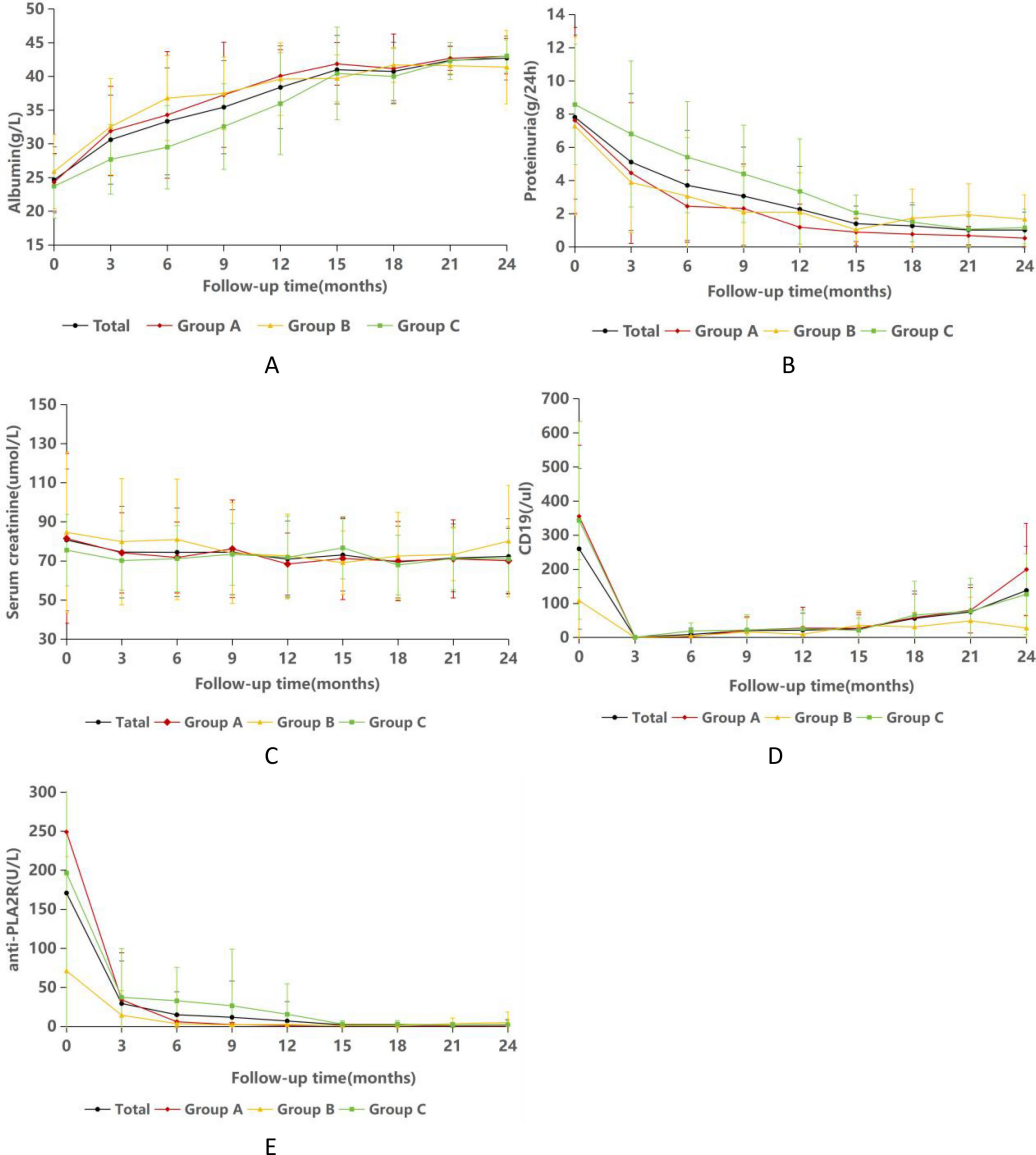
Time course of clinical remission and immunological response to obinutuzumab over 24 months. Serial levels of albumin **(A)**, proteinuria **(B)**, and serum creatinine **(C)**, and the absolute values of CD19 **(D)** and anti-PLA2R antibody **(E)** after the obinutuzumab treatment in patients who were followed up for 24 months. Data are presented as median (interquartile range) over time **(B–E)** or mean ± SD **(A)**. Data are shown descriptively; no statistical comparison was performed over time.

During the 24-month follow-up period, all the PMN patients exhibited an overall downward trend in anti-PLA2R antibodies, 24-h urinary protein quantification, and absolute CD19 values, whereas serum albumin showed an upward trend (for details, see **Figure 3**). The 24-month follow-up results showed that anti-PLA2R antibody levels in all the PMN patients decreased from 50.2 (8.1, 222.3) U/mL to 0.98 (0.45, 1.58) U/mL, with an overall seroconversion to negative. The 24-h urinary protein level decreased from 6.3 (4.0, 9.8) g/24 h to 0.56 (0.23, 1.32) g/24 h. Serum albumin gradually increased from 24.7 ± 4.9 g/L to 43.0 (41.4,44.4) g/L (for details, see **Table 3**). No patients in this study progressed to ESKD. Among the 63 anti-PLA2R antibody-positive patients and 16 anti-PLA2R antibody negativity but no immunological remission (2–20 U/mL) patients, antibody levels decreased in all cases, with 54 (68.4%) achieving seroconversion (<2 U/mL). Among 54 patients with antibody-negative status, 50 (92.6%) achieved clinical remission.

**Table 3:** Clinical characteristics of PMN patients after obinutuzumab treatment for 24 months.

Characteristic	Total	Group A	Group B	Group C	P^a^
Time to reach remission (months)	16.0 (7.8, 26.5)	12.0 (6.0, 19.5)	13.0 (8.0, 26.0)	23.0 (8.0, 40.0)	0.006
Proteinuria (g/24 h)	0.56 (0.23, 1.32)	0.33 (0.19, 0.98)	1.05 (0.33, 3.27)	0.90 (0.38, 1.32)	0.076
Albumin (g/L)	43.0 (41.4, 44.4)	43.1 (42.1, 45.4)	42.7 (40.7, 45.4)	43.1 (41.4, 43.9)	0.779
Anti-PLA2R antibody positivity, n(%)	5 (5.32%)	2 (6.25%)	1 (3.13%)	2 (6.67%)	0.791
Anti-PLA2R antibodies (U/mL)	0.98 (0.45, 1.58)	1.15 (0.47, 1.90)	0.98 (0.44, 1.78)	0.92 (0.52, 1.44)	0.739
Serum creatinine (μmol/L)	65.9 (56.8, 78.8)	65.9 (55.7, 74.2)	67.8 (58.1, 104.8)	66.2 (56.8, 78.2)	0.948
The absolute values of CD19 (/μL)	78.6 (21.4, 249.5)	187.9 (80.0, 331.9)	11.0 (0, 56.8)	76.8 (19.0, 249.5)	0.001

**Table 5:** Univariable and multivariable Cox regression analyses: predictors of clinical remission within 24 months.

Characteristic	Univariate Cox analysis		Multivariate Cox analysis	
	HR (95% CI)	P-value	HR (95% CI)	P^c^
Male sex	0.776 (0.494,1.221)	0.273		
Age (years)	1.010 (0.993,1.028)	0.251		
Urine RBC/µL	0.998 (0.990,1.007)	0.730		
Proteinuria (g/24 h)	0.946 (0.899,0.995)	0.031	0.956(0.908, 1.007)	0.087
WBC (×10^9^/L)	0.975 (0.902,1.054)	0.522		
Haemoglobin (g/L)	1.005 (0.995,1.014)	0.321		
Platelet (×10^9^/L)	0.998 (0.996,1.001)	0.232		
ALT	0.982 (0.951,1.013)	0.257		
AST	0.983 (0.953,1.015)	0.306		
Total protein (g/L)	1.005 (0.979,1.032)	0.718		
Albumin (g/L)	1.015 (0.976,1.056)	0.454		
Globulin (g/L)	0.966 (0.918,1.015)	0.171		
Cholesterol (mmol/L)	0.886 (0.802,0.979)	0.018	0.903(0.817, 0.998)	0.046
Serum creatinine (μmol/L)	1.000 (0.994,1.006)	0.956		
eGFR (mL/min/1.73 m^2^)^a^	1.001 (0.993,1.009)	0.824		
Anti-PLA2R antibodies (U/mL)	1.000 (0.999,1.000)	0.461		
The absolute values of CD19(/μL)	1.000 (0.999,1.001)	0.747		

### Safety analysis

During the treatment with obinutuzumab and the follow-up period in this study, a total of 18 patients (19.15%) experienced adverse events, and five patients (5.32%) developed serious adverse events (two were hospitalised due to herpes zoster and three due to pulmonary infection). The most common adverse reactions were infusion-related reactions, including rash, erythema, itching, runny nose, and restlessness, all of which resolved spontaneously after the infusion was completed. Only the patients with severe herpes zoster and pulmonary infection received systemic treatment, and all the patients achieved full recovery (for details, see **Table 4**).

**Table 4:** Adverse events in all patients with PMN receiving obinutuzumab.

Events	Patients(n)	No. of events(n)
Any adverse event	18	18
Serious adverse events	5	5
Fatal	0	0
Non-fatal	5	5
Fever, pulmonary infection	3	3
Herpes zoster	2	2
Non-serious adverse events	13	13
Infusion reactions*	8	8
Swelling of the limb on the infusion side with joint pain	1	1
Diarrhoea	0	0
Ileus	0	0
Nausea and dizziness with transient tinnitus	2	2
Flustered	1	1
Weight loss	1	1

*Infusion reactions include bronchial wheezing, rash, erythema, itching, rhinorrhoea, and dysphoria.

## Discussion

CTX has long been a standard treatment for MN due to its ability to prevent end-stage renal failure. However, this drug increases the risk of malignancy and carries the potential for irreversible reproductive toxicity (25). Treatment with CNIs, such as CSP or TAC, can achieve remission rates of 60% to 70% but is associated with high relapse rates and nephrotoxicity (25). Recently, with significant advances in the understanding of the pathogenesis of MN, RTX, which targets CD20 on B cells, has been recommended as a first-line therapy in guidelines, and its treatment course is well tolerated (22). However, a subset of patients with PMN does not respond to RTX, with only 60%–70% of patients achieving sustained clinical remission (16, 26–29). Given the limitations of conventional regimens, such as uncertain efficacy and adverse reactions, identifying safe and effective alternative therapies for the treatment of PMN has become a major challenge.

Obinutuzumab, like RTX, is an anti-CD20 antibody. However, it recognises overlapping epitopes of CD20 in a different orientation and is glycoengineered to produce greater cytotoxicity and B-cell depletion capacity through mechanisms such as direct cell death, antibody-dependent phagocytosis, and antibody-dependent cellular cytotoxicity (20, 30). Therefore, obinutuzumab has become an alternative for PMN patients who have failed RTX treatment or cannot tolerate RTX (30, 31). Limited data are available on the use of obinutuzumab in treating PMN patients, but some evidence suggests that obinutuzumab may be effective in treating certain kidney diseases (21, 32–38). This study, through retrospective analysis, further confirms the therapeutic efficacy and safety of obinutuzumab in Chinese PMN patients. The results demonstrate that the vast majority of patients achieved clinical remission, with significantly reduced or seroconverted anti-PLA2R antibody levels, relatively stable renal function, and no patients progressing to ESKD. The study also emphasises the necessity of antibody clearance for achieving and enhancing clinical remission.

The study results showed that the median time to achieve clinical remission in the three groups was 16.0 months overall, 12.0 months in group A, 13.0 months in group B, and 23.0 months in group C (P = 0.006, the difference was statistically significant). The onset of response was slower in group C, which may be attributed to their higher baseline urinary protein levels and a more complex immune network due to prior RTX exposure. Nevertheless, 90% of patients in this group eventually achieved remission, indicating that obinutuzumab is effective in RTX-resistant patients; albeit with a slower onset, it remains effective in the long term. There was no significant difference in remission time between group A and group B, suggesting that switching to obinutuzumab after failure of conventional immunosuppressive therapy does not require additional prolonged waiting.

To overcome the potential impact of imbalanced baseline characteristics on the comparison of efficacy in this retrospective cohort, we further used a Cox proportional hazards regression model to evaluate independent predictors of achieving clinical remission (CR+PR) within 24 months. Univariate analysis showed that baseline 24-h urinary protein levels and total cholesterol were significantly associated with clinical remission. After including these two variables in a multivariate model, total cholesterol remained an independent predictor of remission, whereas the effect of proteinuria diminished. Variables such as sex, age, eGFR, anti-PLA2R antibody levels, and CD19 count did not show significant predictive value (for details, see **Table 5**). These findings suggest that lower baseline cholesterol levels may reflect a relatively milder nephrotic syndrome state, thereby facilitating clinical remission; while proteinuria is associated with remission, its effect may be confounded by other factors. Of note, anti-PLA2R antibody levels were not identified as an independent predictor in this study, which is somewhat inconsistent with the results of some previous rituximab studies, possibly due to the rapid antibody seroconversion after obinutuzumab treatment in most patients in this cohort, as well as the limited sample size.

This study demonstrated that 27 patients (90%) in group C achieved clinical remission, which was lower than the rates in groups A and B (P = 0.118), with no statistically significant difference. However, this result is superior to the 85.7% and 80% clinical remission rates reported by Sethi et al. (34) and Xu et al. (39), respectively. The higher remission rate in group C of this study compared with previous reports may be attributed to differences in patient populations, drug dosages, and follow-up durations. The findings of this study suggest that, compared with historical data, obinutuzumab may have a favourable clinical remission rate in patients with PMN. As a robust supplementary treatment option, obinutuzumab effectively addresses the limitations of first-generation RTX therapy, mitigates the risk of treatment failure induced by first-generation monoclonal antibodies, and enhances the success rate of treatment.

Compared with previous RTX studies, the remission rates in groups A and B in this study were numerically higher. Specifically, the remission rate in group A was 100%, superior to the 69.1% remission rate for RTX initial treatment reported by Ruggenenti et al. (16), the 60% remission rate in the MENTOR study (26), the 62% remission rate in the RI-CYCLO study (40), the 64.9% remission rate in the EMRITUX study (15), and the 88.9% remission rate in our group’s earlier study (41). The remission rate in group B was 96.88%, outperforming the 50.0% remission rate reported by Ruggenenti et al. (42), the 41.7% clinical remission rate reported in a study by Peking University First Hospital (27), and the 66.7% remission rate in our group’s earlier study (41). In group C, three patients (10%) did not achieve remission after 2 years of follow-up following the completion of the full-dose obinutuzumab injections. However, within the limited follow-up period, their proteinuria, anti-PLA2R antibody levels, and absolute CD19 values showed a declining trend. It is suspected that prolonged and heavy proteinuria may have led to chronic kidney injury in these patients. Alternatively, there is a possibility that they later developed focal segmental glomerulosclerosis (FSGS). Considering the patients’ actual circumstances and clinical factors, a repeat renal biopsy was not performed, and the exact reasons remain unclear.

In terms of adverse events, the majority of patients in this study tolerated obinutuzumab well, with adverse and serious adverse events occurring in 19.15% and 5.32% of the patients, respectively. Additionally, while the most common adverse reactions in previous studies were infusion-related, such reactions were relatively infrequent in this study. This infrequency may be attributed to the use of premedication with antihistamines and controlled infusion rates, which likely helped prevent or mitigate infusion-related events to some extent. Regarding serious adverse events, a thorough review of the relevant data in this study confirmed that none were related to malignancies or life-threatening conditions. This study further supports the acceptable short- to medium-term safety profile of obinutuzumab in the treatment of PMN. Long-term safety still needs to be confirmed by large-scale prospective studies.

Our study is a retrospective observational analysis based on real-world data, which has certain limitations and experimental biases. (1) This is a single-centre, retrospective, non-randomised controlled study, which is subject to selection bias and recall bias. Patients in each group were not randomly assigned but were classified *post-hoc* based on treatment history, leading to significant differences in baseline characteristics (e.g., proteinuria, anti-PLA2R antibody levels, CD19 count). (2) Although we used multivariate Cox regression to adjust for some confounding factors, due to the limited sample size (n=94) and the relatively moderate number of remission cases (n=90), we were unable to include all potential confounding variables, nor did we perform propensity score matching or instrumental variable analysis. (3) Regarding the longitudinal changes in proteinuria, antibody levels, etc., shown in **Figure 3**, we only performed graphical descriptions without using repeated-measures statistical methods such as linear mixed models. Despite these limitations, our research lays the foundation for future large-scale prospective studies to further investigate the application of obinutuzumab in PMN.

In conclusion, this study demonstrates that obinutuzumab has a high clinical remission rate and acceptable short-term safety in patients with PMN, providing a potentially effective treatment option for those with refractory disease or poor response to rituximab. Rational and standardised administration, along with regular monitoring of anti-PLA2R antibody levels during treatment, may help improve therapeutic outcomes and reduce adverse events.

## Data Availability

The data supporting the findings of this study are openly available in Mendeley Data at DOI: 10.17632/8mdcpfkdrc.1.
